# Whole Mitogenomes Reveal the History of Swamp Buffalo: Initially Shaped by Glacial Periods and Eventually Modelled by Domestication

**DOI:** 10.1038/s41598-017-04830-2

**Published:** 2017-07-05

**Authors:** S. Wang, N. Chen, M. R. Capodiferro, T. Zhang, H. Lancioni, H. Zhang, Y. Miao, V. Chanthakhoun, M. Wanapat, M. Yindee, Y. Zhang, H. Lu, L. Caporali, R. Dang, Y. Huang, X. Lan, M. Plath, H. Chen, J. A. Lenstra, A. Achilli, C. Lei

**Affiliations:** 1 College of Animal Science and Technology, Northwest A&F University, Yangling, Shaanxi 712100 China; 2 Dipartimento di Biologia e Biotecnologie “L. Spallanzani”, Università di Pavia, Pavia, 27100 Italy; 3 School of Bioscience and Engineering, Shaanxi University of Technology, Hanzhong, Shaanxi 723000 China; 4 Dipartimento di Chimica, Biologia e Biotecnologie, Università di Perugia, Perugia, 06123 Italy; 5 Key Laboratory of Plateau Lake Ecology and Global Change, College of Tourism and Geography, Yunnan Normal University, Kunming, Yunnan 650500 China; 6 Faculty of Animal Science and Technology, Yunnan Agricultural University, Kunming, Yunnan 650201 China; 7 Department of Animal Science, Faculty of Agriculture and Forest Resource, Souphanouvong University, Luang Prabang, Laos; 8 Tropical Feed Resources Research and Development Center, Department of Animal Science, Faculty of Agriculture, Khon Kaen University, Khon Kaen, 40002 Thailand; 9 Department of Clinical Science and Public Health, Faculty of Veterinary Science, Mahidol University, Kanchanaburi campus, Kanchanaburi, 71150 Thailand; 10 National Engineering Laboratory for Animal Breeding, Key Laboratory of Aniaml Genetics and Breeding and Reproduction of MOA, College of Animal Science and Technology, China Agricultural University, Beijing, 100193 China; 11 IRCCS Institute of Neurological Sciences of Bologna, Bologna, 40139 Italy; 12 Faculty of Veterinary Medicine, Utrecht University, Yalelaan 104, 3584 CM Utrecht, The Netherlands

## Abstract

The newly sequenced mitochondrial genomes of 107 Asian swamp buffalo (*Bubalus bubalis carabensis*) allowed the reconstruction of the matrilineal divergence since ~900 Kya. Phylogenetic trees and Bayesian skyline plots suggest a role of the glacial periods in the demographic history of swamp buffalo. The ancestral swamp-buffalo mitogenome is dated ~232 ± 35 Kya. Two major macro-lineages diverged during the 2^nd^ Pleistocene Glacial Period (~200–130 Kya), but most (~99%) of the current matrilines derive from only two ancestors (SA1′2 and SB) that lived around the Last Glacial Maximum (~26–19 Kya). During the late Holocene optimum (11–6 Kya) lineages differentiated further, and at least eight matrilines (SA1, SA2, SB1a, SB1b, SB2a, SB2b, SB3 and SB4) were domesticated around 7–3 Kya. Haplotype distributions support an initial domestication process in Southeast Asia, while subsequent captures of wild females probably introduced some additional rare lineages (SA3, SC, SD and SE). Dispersal of domestic buffaloes created local population bottlenecks and founder events that further differentiated haplogroup distributions. A lack of maternal gene flow between neighboring populations apparently maintained the strong phylogeography of the swamp buffalo matrilines, which is the more remarkable because of an almost complete absence of phenotypic differentiation.

## Introduction

Water buffalo (*Bubalus bubalis*) is one of the most important livestock species in several Asian countries and is used for the production of milk and meat and for draft power in rice cultivation. The domestic water buffalo in Asia is generally divided in two major subspecies, the dairy river buffalo and the draft swamp buffalo, which differ in morphology, behavior and number of chromosomes^1, 2^. The river buffalo is found in the Indian subcontinent, South Asia and the Mediterranean area (Italy, Egypt and the Balkans), and sporadically in Australia and South America, whereas the swamp buffalo is kept in Northeast India, China (southern regions and Yangtze valley) and Southeast Asia^1, 3^. Both types of water buffalo descend from the wild Asian buffalo (*Bubalus arnee*)^4^, which had a widely distribution range in eastern Indian, Sri Lanka and Southeast Asia until the beginning of XIX century^5–8^. Lau *et al*.^7^ hypothesized that the wild Asian buffalo originated in mainland of Southeast Asia and spread north toward China and west toward the Indian subcontinent, where the river type was probably domesticated.

Mitochondrial DNA (mtDNA), Y-chromosomal and nuclear microsatellite data showed a deep genetic divergence of swamp and river buffalo^2, 5–7, 9–16^, which indicate two independent domestications^5, 17^. Domestication of river buffalo most likely took place in the Indian subcontinent^15^, whereas swamp buffalo was proposed to originate from the border region between south China and north Indochina^6, 17^. However, the fragmentary buffalo mtDNA sequences from rDNA, COII, and *cytochrome b* loci reported to date^5–7, 18^, confound quantitative inferences of population history. So far two swamp buffalo mitogenomes (NC006295/AY702618 and JN632607) and one river buffalo mitogenome (AF547270) were deposited in GenBank. In this study, we report the mitogenomes from an additional 107 Southeast-Asian swamp buffaloes, covering most of its current geographic distribution with as outgroup one mitogenome from a Chinese river buffalo in order to establish the haplogroup phylogeny and reconstruct the demographic history of the swamp buffalo.

## Results

### Sequence variation of swamp buffalo mitogenomes

The 109 swamp buffalo mitogenomes with a length of 16340 to 16363 bps belong to 87 different haplotypes (Ht.s) and are divided into 21 haplogroups or subhaplogroups (Supplementary Dataset S1). The swamp haplotypes (Hd: 0.992) contain 362 polymorphic sites (π: 0.422) with a pairwise nucleotide difference of 69.0 ± 16.5 and a synonymous/non-synonymous ration of 4.63. Similar values have been reported for other livestock and human mitochondrial genomes^19–21^. Seventy-five swamp haplotypes are observed once, while the most frequent haplotypes HT22 and HT55 occurred five and seven times, respectively. Forty-eight haplotypes with 59 sequences belong to lineage SA and 34 haplotypes with 44 sequences belong to lineage SB. The remaining 5 haplotypes belong to the rare haplogroups SC (2), SD (2) or SE (1). The two river buffalo mitogenomes belong to clades R1 and R2^1, 6^.

### Swamp Buffalo Mitochondrial Phylogeny

A maximum parsimony (MP) tree based on the 111 water buffalo mitogenomes (89 haplotypes) confirms two distinct branches, river and swamp buffalo, which were separated by 300 substitutions (Supplementary Dataset S2). The river buffalo branch includes 2 haplotypes belonging to lineages R1 and R2. The remaining 87 swamp buffalo haplotypes cluster into five divergent haplogroups (Hg.s), namely SA, SB, SC, SD and SE, with an overwhelming representation of SA (54.1%) and SB (40.4%). We largely confirm previous phylogenies^6, 10^, but also reveal the novel haplogroups SA3 and SB4 and many different subhaplogroups. Lineages SA and SB are divided into three (SA1 to SA3; SA1′2 as an ancestral node) and four (SB1-SB4; SB2′3′4 as an ancestral node) sublineages, respectively. A single control-region transition at position 16066 defines a major (32.1%) star-like subclade SA1a. For the three rare lineages SC (1.8%), SD (2.8%) and SE (0.9%), the complete mtDNA sequences confirm that the split-off of lineage SC preceded the SA-SB divergence and that SD is a sister clade of SB, but indicate that also SE and SA are sister clades. Maximum likelihood (ML) and Bayesian evolutionary analysis of sampling trees (Beast) retrieved remarkably similar tree topologies (Supplementary Figs S1 and S2).

### Molecular Clocks and Age Estimates

Previously reported estimates of the divergence time between river and swamp range from 10 Kya to 1.7 Mya^2, 5, 7, 9–11, 13–16, 18, 22, 23^, partially because different mtDNA segments were analyzed and different evolution rates were applied. In the present study, we phylogenetically compared 111 water buffalo mitogenomes with one African buffalo (*Syncerus caffer*; NC020617), while rooting our MP tree with one *Bos taurus* (V00654.1) and one ancient *Bos primigenius* (GU985279) mitogenome. The divergence pattern evaluated on all 114 bovine mitogenomes (Supplementary Fig. S3) confirms saturation of the D-loop divergence, emphasizing that sequencing of whole mitogenomes is essential for quantification.

**Figure 1:**
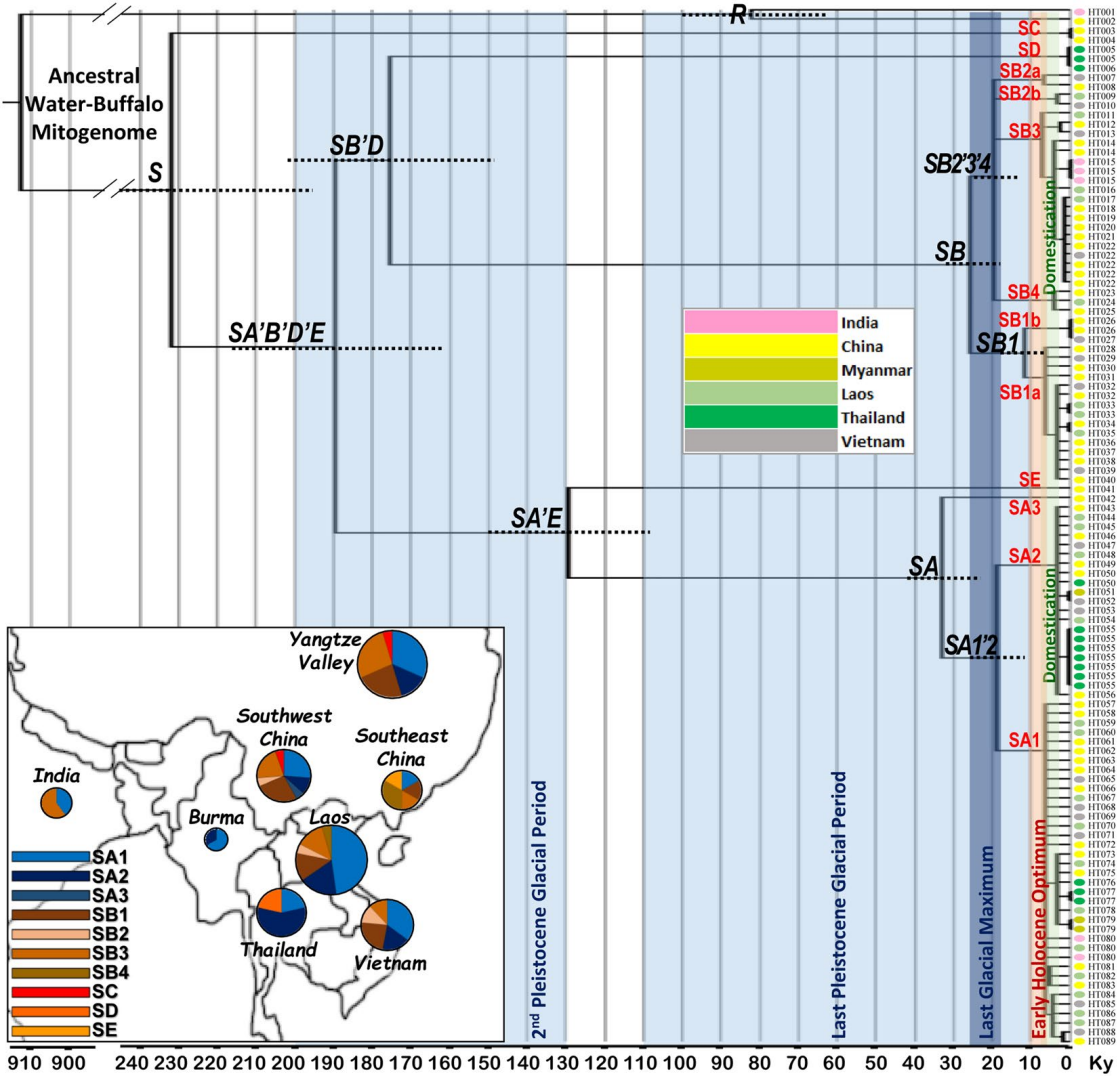
Phylogeny of complete mtDNAs from 111 buffalo mitogenomes. The topology was inferred by maximum parsimony (Supplementary Dataset S2). A maximum likelihood time scale, based on synonymous substitutions, is indicated below the tree. The standard error for the major nodes are represented by dot lines, further details are available in Table 1. Samples are indicated by 89 different haplotype IDs (Supplementary Dataset S1). The insert shows the geographic distribution of major haplogroups based on these complete mtDNAs. Samples from China have been divided into three regions (Yangtze Valley, Southwest China and Southeast China). The map has been drawn by hand in Adobe Photoshop (v. 8.0; http://www.adobe.com).

We first considered the synonymous mutations for a ML estimation of the molecular age of phylogenetic nodes (Fig. 1). Using a fossil age estimate of the *Bovini* tribe of 8.8 Mya^24^ and the age (6.7 Ky) of the ancient *Bos primigenius* mtDNA^25^ we calculated a rate of 3.75 ± 0.47 × 10^−5^ synonymous substitutions per nucleotide per Ky for 3790 amino acid codons equalling 1 synonymous substitution every ~7.030 Ky. This yields divergence times of ~913 ± 78 Kya for the water buffalo mitogenome, ~82 ± 18 Kya for the only two river buffalo R1 and R2 haplotypes and ~232 ± 35 Kya for the swamp buffalo haplogroups (Table 1 and Fig. 1). At least 12 different mtDNA ancestral haplotypes of the current swamp buffaloes were present in Southeast Asia during the early Neolithic (11–6 Kya), which overlaps with the initial phase of domestication^26^. These haplotypes were the ancestors of the eight (sub)haplogroups SA1, SA2, SB1a, SB1b, SB2a, SB2b, SB3 and SB4, still common in modern herds, and of the rare SA3, SC, SD and SE.

**Table 1:** Age estimates of major buffalo branches based on different mitochondrial datasets.

Node	N	ML (synonymous subst)		ML (only coding region)		ML (all substitutions)^a^		Beast (all substitutions)^a^	
		T(ky)	SE^b^(ky)	T(ky)	SE^b^(ky)	T(ky)	SE^b^(ky)	T(ky)	SE^b^(ky)
**A.W.M**.^**c**^	111	912.6	78.3	1044.3	71.4	721.6	59.1	672.0	101.7
**River**	2	81.9	18.3	109.3	20.7	68.6	11.6	63.7	13.2
**Swamp**	109	231.8	35.3	280.1	28.8	204.4	20.0	194.2	31.4
**SC**	2	0.0	59.1	3.8	3.8	1.5	1.5	2.9	1.4
**SA**′**B**′**D**′**E**	107	189.6	27.2	222.9	25.0	176.6	17.7	166.6	26.8
**SA**′**E**	60	129.3	21.2	152.7	20.9	129.1	15.5	120.6	20.7
**SE**	1	n.a.	n.a.	n.a.	n.a.	n.a.	n.a.	n.a.	n.a.
**SA**	59	33.0	9.2	40.5	9.9	37.5	7.3	35.9	7.9
**SA1**′**2**	56	18.8	6.5	23.9	7.4	18.4	4.5	18.5	4.6
**SA1**	37	6.4	4.2	8.1	5.4	7.2	1.8	8.4	2.2
**SA1a**	35	6.4	1.5	8.1	1.7	5.5	0.9	6.7	1.5
**SA1a1**	3	5.2	1.6	6.8	1.8	4.8	1.0	4.1	1.2
**SA1a2**	6	4.4	1.7	5.7	1.9	4.5	1.1	4.2	1.0
**SA1a3**	9	3.3	2.4	4.4	2.5	3.9	1.2	4.2	1.0
**SA2**	21	3.3	1.5	3.6	1.6	5.1	1.2	7.0	1.8
**SA3**	1	n.a.	n.a.	n.a.	n.a.	n.a.	n.a.	n.a.	n.a.
**SB′D**	47	175.5	27.1	210.0	25.4	157.7	16.9	146.4	24.3
**SD**	3	0.0	44.6	0.0	44.8	0.9	1.0	3.2	1.3
**SB**	44	25.8	7.0	35.1	8.3	30.7	5.9	31.1	6.8
**SB1**	18	11.4	6.2	12.2	4.9	8.6	2.4	8.9	2.7
**SB1a**	15	6.4	4.4	8.0	2.7	5.8	1.4	6.0	1.4
**SB1a1**	11	3.3	2.0	4.2	2.1	3.7	1.2	4.5	1.0
**SB1a2**	2	6.4	4.5	6.5	2.4	4.9	1.4	3.8	1.0
**SB1b**	3	0.0	13.0	0.0	13.1	1.2	1.4	3.5	1.3
**SB2**′**3**′**4**	26	19.8	5.7	26.1	6.7	23.3	4.7	23.4	5.3
**SB2**	4	19.8	10.6	26.1	12.5	15.7	4.5	10.8	4.0
**SB2a**	2	3.1	3.1	6.7	4.8	6.0	2.9	3.9	1.4
**SB2b**	2	6.8	6.8	7.0	7.0	9.9	4.5	5.2	2.3
**SB3**	19	6.9	3.4	7.2	3.5	4.7	1.8	6.6	1.7
**SB3a**	16	4.1	3.1	4.6	3.0	2.4	1.0	5.0	1.1
**SB3a1**	10	1.2	0.9	1.8	1.1	1.4	0.6	3.9	0.8
**SB4**	3	4.1	2.9	8.7	4.2	5.1	2.2	4.7	1.7

^a^The entire genome was partitioned into coding and control region.

^b^The 95% Confidence Interval (CI) corresponds to 1.96 times the value of the Standard Error (SE) reported here.

^c^Ancestral Water-Buffalo Mitogenome.

Time estimates were confirmed by (i) using all open reading frame mutations, (ii) considering all mutations partitioned in coding and control regions and evaluated with both ML and Beast (Table 1). However, slightly deleterious mutations within the open reading frames may lead to overestimations of younger clades (Supplementary Fig. S4)^19, 21^. The overall mutation rate was estimated at 2.11 ± 0.34 × 10^−8^ substitutions per nucleotide per year (1 mutation every ~2900 years) over the entire mitogenome. As for the river buffalo internal variation, a better estimate will be obtained by analyzing more mitogenomes.

### Estimating Past and Present Demographic Trends

**Figure 2:**
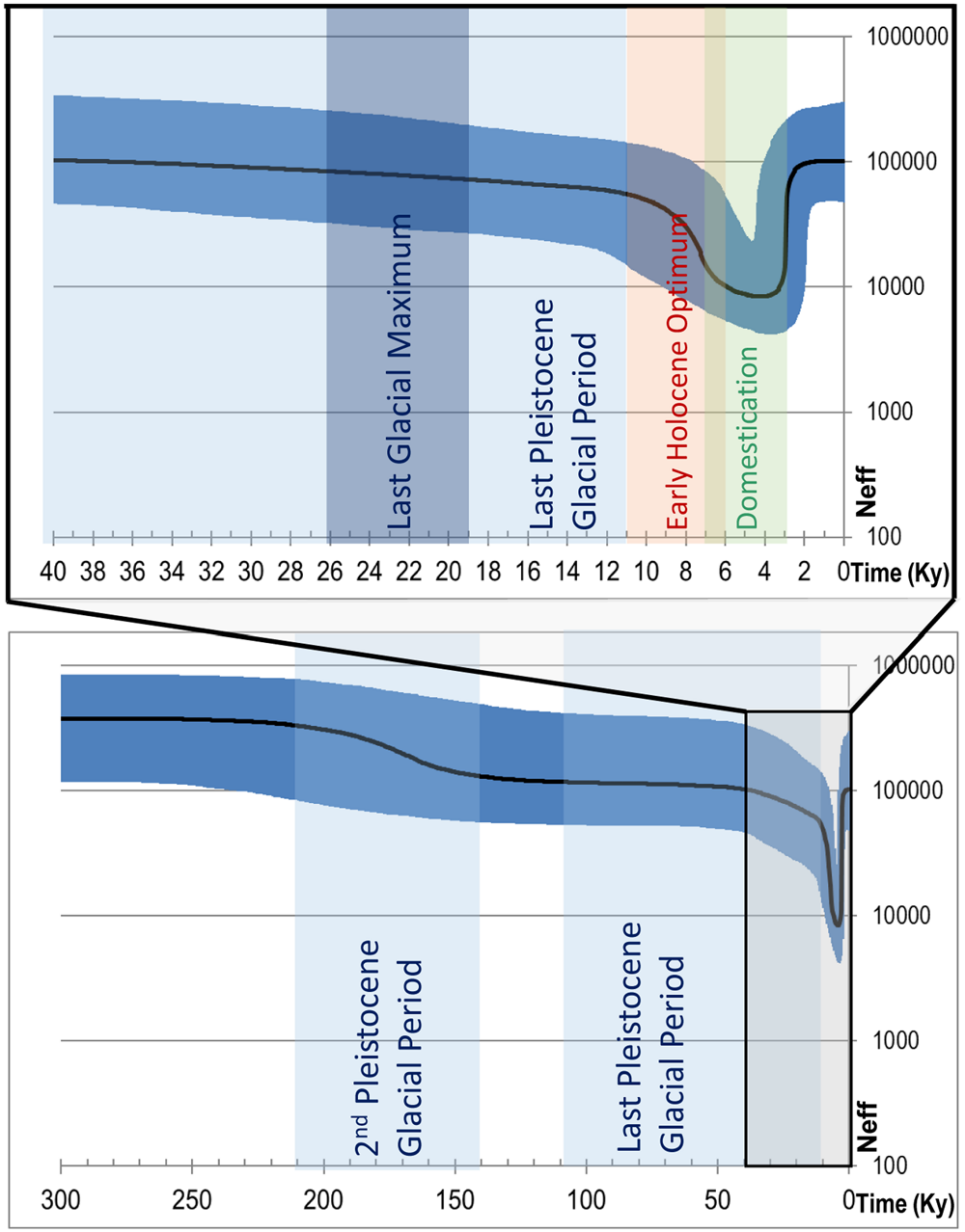
Bayesian skyline plot showing the swamp buffalo population size trend. The Y axis indicates the effective number of females, as inferred from our mitogenome dataset considering a generation time of six years^49^. The black solid line is the median estimate and the blue shading shows the 95% highest posterior density limits.

Bayesian skyline plot (BSP) of swamp buffalo mitogenomes shows three major changes in the effective female population size: i) a slight decrease between about 200 and 130 Kya; ii) a more recent decrease starting around 25–20 Kya and much steeper during the early Neolithic (11–6 Kya); and iii) a rapid increase from 3 Kya (Fig. 2). This recent increase explains the star-like topology of some haplogroups (e.g. SA1, SA2, SB1a, SB3 and SB4; Fig. 1) and is very similar to previously analysis of water buffalo and other bovine domestic species^27^.

An analysis of the geographic distribution of swamp haplogroups in Southeast Asia, based on the control-region data currently available in previous literature^6^ or deposited in GenBank (Supplementary Table S1) confirms the prevalence of SA or SB mtDNAs (>99%) and a geographic differentiation of subhaplogroups with contrasting geographic distributions of the subhaplogroups SA2, SB1, SB2 and SB3 (Supplementary Fig. S5). The rare haplogroup SC was previously reported to occur in Thailand, Bangladesh and sporadically in Southwest China, while SD and SE were found only in Thailand^6^. We confirmed the presence of SC in the Southwestern Chinese Dehong population, but the haplotypes SC and SE were also found in the Yibin and Poyanghu breeds from the Yangtze Valley (inset in Fig. 1).

## Discussion

We sequenced the complete mitogenome of the swamp buffalo in order to reconstruct the phylogenetic relationships of the mitochondrial haplotypes and to obtain a time scale for the phylogeny. Most of the major haplogroups were already identified in previous studies^2, 5, 7, 9–11, 13–16, 18, 22, 23^, but we recognized the novel haplogroups SA3 and SB4 and defined the subhaplogroups SA1a, SA1a1, SA1a2, SA1a3, SB1a, SB1a1, SB1a2, SB1b, SB2a, SB2b, SB3a, SB3a1, SD1 and SD2 (Supplementary Dataset S1 and S2).

During the glacial periods, drastic changes in ecological and climatic seasons had consecutive major effects on the distribution of plants and animals^28, 29^. As part of the Indo-Pacific Warm Pool, Southeast Asia was relatively warm during the Last Glacial Maximum (~26–19 Kya)^30^ with a temperature decrease of only 2.5 °C *vs* 5–10 °C globally^31, 32^. This made Southeast Asia a major global biodiversity hotspot^33^ in which many species survived (in glacial refuges) the fluctuations of temperature and forest coverage during the Pleistocene (for the latter, see Supplementary Fig. S6)^28^.

Estimations of divergence times are inherently imprecise^34^, but the accuracy of our data has been optimized by using data from the aurochs as internal calibration point, this in addition to a fossil age of the *Bovini* tribe of 7–11 Mya^24^. According to our estimate, the divergence of the swamp and river types of the water buffalo took place almost at the beginning of a glacial period (~900 to 860 Kya). Remarkably, from the swamp matrilineal diversity that must have formed until 200 Kya only the minor haplogroups SC and the ancestor of all other haplogroups (SA′B′D′E) have survived. We propose to divide the last 200 ka into five phases, correlating glacial periods and estimates of demographic/phylogenetic history (Figs 1 and 2 and Supplementary S6).

1. During 2^nd^ Pleistocene Glacial Period (~200 to 130 Kya) the first decline of population was observed in the BSP while two macro-haplogroups (SA′E and SB′D) diverged (Figs 1 and 2).
2. The first phase of the last Pleistocene glacial period (~110 to 50 Kya) was still comparatively moderate, the population size remained almost unchanged and only one divergence event (SA-SE) has been identified.
3. During the second phase of the last Pleistocene glacial period (~50 to 11 Kya) the population began to decline. The current demographic composition of swamp populations shows that ~99% of current mitogenomes are derived from only two ancestral haplotypes (SA1′2 and SB), both dated around the LGM (~26–19 Kya). Afterwards, the major haplogroups SA1′2 and SB differentiated into 8 haplotypes (SA1, SA2, SB1a, SB1b, SB2a, SB2b, SB3 and SB4).
4. After 11 Kya the increasing temperature raised the sea level, which had a profound impact in the regions from Sundaland to Southeast China^35^. Paleoenvironmental data on the Holocene indicate a warm period between 11 and 6 Kya in southern China, known as the early Holocene optimum^36^. This overlapped with the first phases of the rice cultivation, which is believed to have triggered the domestication of the swamp buffalo at 7–3 Kya and an expansion of the water buffalo population^1, 6, 10, 14, 26, 37^.
5. Finally, we observe a rapid increase since 3 Kya to the present population size. As previously proposed^27^, this was due to expansion of the domestic buffalo to the large current distribution range, harboring the several present populations with distinct haplogroup distributions.

Thus, the demographic history of swamp buffalo seems to be linked to the historic glacial events, establishing isolated refugia of swamp buffalo in which only a limited number of subhaplogroups survived. This may explain why only one divergence event (SA-SE) has been dated to the period of 180–40 Kya. The current demographic composition of swamp populations suggests that ~99% of current mitogenomes are derived from only two ancestors (SA1′2 and SB), which are both dated around the Last Glacial Maximum (~26–19 Kya). The time estimates in Fig. 1 and Table 1 further indicate that also the divergence of the current domestic subhaplogroups SA1, SA2, SB1a, SB1b, SB2a, SB2b, SB3 and SB4 preceded domestication, which gives 8 haplotypes as a minimum estimate of the swamp buffalo diversity captured by the first farmers.

Our account of the pre-domestic history of the swamp buffalo provides a context to previous studies of the diversity of mtDNA control region and cytochrome *b* gene^6, 10^, which has been summarized in Supplementary Fig. S5. The high diversity of domestic SA and SB haplotypes in the China-Vietnam border region was proposed as evidence supports an initial major domestication event of swamp buffalo in Southeast Asia, probably between southern China and Vietnam. The finding of SC, SD and SE haplotypes almost exclusively in Thailand and Bangladesh suggests incorporation of these haplotypes after the domestic buffaloes had reached the west bank of the Mekong river^6^ in a scenario of recurrent restocking the domestic population with wild females as proposed previously for the horse^20, 38, 39^. Extending the analysis to other loci or even to the entire genome and also a wider sampling covering the entire geographic range of the swamp buffalo is desirable to unravel further the domestication and subsequent demographic history of swamp buffalo and to enable an interesting comparison with the related river buffalo^6^.

## Methods

### Sample Collection

Most of the samples used for this work were already collected from previous collaborative works^5, 10^. All samples were already classified into mtDNA haplogroups based on control-region data. We selected 107 mtDNA for complete sequencing in order to represent all swamp lineages and to include the highest possible molecular variability avoiding potential redundancies. An additional mitogenome from river buffalo was sequenced to be used as an outgroup.

### Mitogenome sequencing

DNA was extracted^10^ from 107 swamp buffaloes (blood, ear tissue and hair follicle) from China (46), Laos (23), Myanmar (3), Thailand (14), Vietnam (16) and India (5) and one Chinese river buffalo. Complete mitogenome sequences were obtained by using two different approaches: 1) PCR amplification (with 27 primer pairs, Supplementary Table S2A)^40^ and Sanger sequencing; 2) Long-Range PCR amplification (Supplementary Table S2B) and Illumina sequencing^41^. GenBank accession number, sequencing method, coverage and depth of each sample are reported in Supplementary Dataset S1. MtDNA genome sequences were analyzed using DNASTAR 7.0, Sequencher v5, DNAsp v5, Clustal X and GeneSyn packages.

All experimental procedures were performed in accordance with the Regulations for the Administration of Affairs Concerning Experimental Animals approved by the State Council of People’s Republic of China. The study was approved by Institutional Animal Care and Use Committee of Northwest A&F University (Permit Number: NWAFAC1019).

### Phylogeny Construction and Demographic Inferences

The phylogeny construction was performed following a maximum parsimony (MP) criterion by hand and confirmed using an adapted version of mtPhyl4.015^42^, as previously described^20, 21, 43, 44^. The modified.txt files to be loaded in the program are available upon request. The tree was rooted on the *Bos taurus* reference sequence (V00654.1) and on the ancient *Bos primigenius* mtDNA (GU985279). A maximum likelihood (ML) tree was computed using MEGA7.0^45^ with 1000 bootstrapping replicates.

A first ML analysis was performed using PAML X^46^ by considering only synonymous mutations in the protein coding genes. The ND6 gene was reverse-complemented to present the same reading direction as the other genes and non-synonymous substitutions were replaced with the ancestral base pairs. Stop codons were excluded from the analysis. Total lengths of coding genes were joined together and the final alignment (11370 bps/3790 codons long) was analyzed with CODEML to calculate a synonymous mutation rate. A second tree was calculated in the same way, but considering all coding mutations. The third and fourth trees with molecular ages were calculated by PAML X and BEAST v. 1.8.3 software^47^, respectively, while considering two partitions in the molecule corresponding to the coding (including all genes coding for mRNA, rRNA and tRNA) and control regions. Modelgenerator v.85 indicated for our dataset HKY + G + I as the best-supported model according to the AIC2 and BIC criterions. These substitution and site heterogeneity models with 8 gamma categories – the lowest number significantly increasing (>1.0) the likelihood – were selected for the subsequent ML and BEAST estimates. The generalized likelihood ratio statistic was always used to verify the clock hypothesis. In order to calibrate the molecular clock, we built a *Bovini* tree by including one African Buffalo (NC020617) and two *Bos* mitogenomes (one *Bos taurus*, V00654; one ancient *Bos primigenius*, GU985279) used as an outgroup. For the calibration point we used the estimated archaeological age of the *Bovini* tribe (8.8 ± 1.1 My; 95% CI: 7–11 My)^24^. Since multiple calibration points are preferable^24^, the age of the ancient *Bos primigenius* (6.7 ± 0.2 Ky; 95% CI: 6.3–7.1 ky) was also used as an internal (recent) calibration point. The major haplogroups were considered as monophyletic in order of being able to calculate their age estimates. The analyses were also repeated excluding the aurochs sequence, but the estimates changed by only ~4% on average. We then obtained a Bayesian skyline plot (BSP)^48^ from the swamp buffalo phylogeny by running 50,000,000 iterations with samples drawn every 10,000 steps. We constructed spatial frequency distribution plots with the program Surfer 9 (Golden Software, http://www.goldensoftware.com/products/surfer) by using the control-region data currently available in previous literature^6^ or deposited in GenBank (Supplementary Table S1).

### Data accessibility

Sequences of the novel water buffalo mitogenomes have been deposited in GenBank under accession numbers KX758295 - KX758402 (108 complete mtDNAs).

## Electronic supplementary material

Supplementary Figures S1-S6 and Tables S1-S2

Supplementary Dataset S2

Supplementary Dataset S1

## References

[1] Cockrill WR (1981). The water buffalo: a review. Br. Vet. J..

[2] Kumar S (2007). Mitochondrial DNA analyses of Indian water buffalo support a distinct genetic origin of river and swamp buffalo. Anim. Genet..

[3] FAO. http://dad.fao.org/ (2014).

[4] Cockrill, W. R. The husbandry and health of the domestic buffalo. (Food and agricultural organization of the United nations, Rome 1974).

[5] Lei CZ (2007). Independent maternal origin of Chinese swamp buffalo (*Bubalus bubalis*). Anim. Genet..

[6] Zhang Y (2016). Strong and stable geographic differentiation of swamp buffalo maternal and paternal lineages indicates domestication in the China/Indochina border region. Mol. Ecol..

[7] Lau CH (1998). Genetic diversity of Asian water buffalo (*Bubalus bubalis*): mitochondrial DNA D-loop and cytochrome b sequence variation. Anim. Genet..

[8] Pandya P (2010). Bacterial diversity in the rumen of Indian Surti buffalo (*Bubalus bubalis*), assessed by 16S rDNA analysis. J. Appl. Genet.

[9] Mishra BP (2015). Genetic analysis of river, swamp and hybrid buffaloes of north-east India throw new light on phylogeography of water buffalo (*Bubalus bubalis*). J. Anim. Breed. Genet..

[10] Yue X-P (2013). Phylogeography and Domestication of Chinese Swamp Buffalo. PLoS One.

[11] Barker JSF (1997). Genetic diversity of Asian water buffalo (*Bubalus bubalis*): microsatellite variation and a comparison with protein-coding loci. Anim. Genet..

[12] Navani N, Jain PK, Gupta S, Sisodia BS, Kumar S (2002). A set of cattle microsatellite DNA markers for genome analysis of riverine buffalo (*Bubalus bubalis*). Anim. Genet..

[13] Barker JSF, Tan SG, Selvaraj OS, Mukherjee TK (1997). Genetic variation within and relationships among populations of Asian water buffalo (*Bubalus bubalis*). Anim. Genet..

[14] Kierstein G (2004). Analysis of mitochondrial D-loop region casts new light on domestic water buffalo (*Bubalus bubalis*) phylogeny. Mol. Phylogenet. Evol..

[15] Kumar S, Nagarajan M, Sandhu JS, Kumar N, Behl V (2007). Phylogeography and domestication of Indian river buffalo. BMC Evol. Biol.

[16] Lei C (2007). Two maternal lineages revealed by mitochondrial DNA D-loop sequences in Chinese native water buffaloes (*Bubalus bubalis*). Asian-australas. J. Anim. Sci.

[17] Yindee M (2010). Y-chromosomal variation confirms independent domestications of swamp and river buffalo. Anim. Genet..

[18] Amano T, Miyakoshi Y, Takada T, Kikkawa Y, Suzuki H (1994). Genetic variants of ribosomal DNA and mitochondrial DNA between swamp and river buffaloes. Anim. Genet..

[19] Soares P (2009). Correcting for purifying selection: an improved human mitochondrial molecular clock. Am. J. Hum. Genet..

[20] Achilli A (2012). Mitochondrial genomes from modern horses reveal the major haplogroups that underwent domestication. Proc. Natl. Acad. Sci. USA.

[21] Colli L (2015). Whole mitochondrial genomes unveil the impact of domestication on goat matrilineal variability. BMC Genomics.

[22] Tanaka K (1995). Nucleotide diversity of mitochondrial DNAs between the swamp and the river types of domestic water buffaloes, *Bubalus bubalis*, based on restriction endonuclease cleavage patterns. Biochem. Genet..

[23] Tanaka K (1996). Phylogenetic relationship among all living species of the genus *Bubalus* based on DNA sequences of the cytochromeb gene. Biochem. Genet.

[24] Bibi F (2013). A multi-calibrated mitochondrial phylogeny of extant Bovidae (Artiodactyla, Ruminantia) and the importance of the fossil record to systematics. BMC Evol. Biol.

[25] Edwards CJ (2010). A complete mitochondrial genome sequence from a mesolithic Wild Aurochs (*Bos primigenius*). PLoS One.

[26] Patel, A. K. & Meadow, R. H. In *Archaeology of the near east*: *Proceedings of the third international symposium on the archeozoology of the southwestern Asia and adjacent areas* (eds H. L. Buitenhuis, L. Bartosiewicz, & A. M. Choyke) (ARC- publications, 1998).

[27] Finlay EK (2007). Bayesian inference of population expansions in domestic bovines. Biol. Lett.

[28] Woodruff DS (2010). Biogeography and conservation in Southeast Asia: how 2.7 million years of repeated environmental fluctuations affect today′s patterns and the future of the remaining refugial-phase biodiversity. Biodivers. Conserv..

[29] De Deckker P, Tapper NJ, van der Kaars S (2003). The status of the Indo-Pacific Warm Pool and adjacent land at the Last Glacial Maximum. Glob. Planet. Change.

[30] Clark PU (2012). Global climate evolution during the last deglaciation. Proc. Natl. Acad. Sci. USA.

[31] Yan X-H, Ho C-R, Zheng Q, Klemas V (1992). Temperature and Size Variabilities of the Western Pacific Warm Pool. Science.

[32] Crowley JT (2000). CLIMAP SSTs re-revisited. Clim. Dyn.

[33] Cannon C (2015). The Ecology of Tropical East Asia by Richard T. Corlett. Q. Rev. Biol..

[34] Graur D, Martin W (2004). Reading the entrails of chickens: molecular timescales of evolution and the illusion of precision. Trends Genet..

[35] Pelejero C, Kienast M, Wang L, Grimalt JO (1999). The flooding of Sundaland during the last deglaciation: imprints in hemipelagic sediments from the southern South China Sea. Earth Planet. Sci. Lett..

[36] Zhou W (2004). High-resolution evidence from southern China of an early Holocene optimum and a mid-Holocene dry event during the past 18,000 years. Quat. Res.

[37] Nagarajan M, Nimisha K, Kumar S (2015). Mitochondrial DNA variability of domestic river buffalo (*Bubalus bubalis*) populations: Genetic evidence for domestication of river buffalo in Indian Subcontinent. Genome Biol. Evol.

[38] Librado P (2016). The evolutionary origin and genetic makeup of domestic horses. Genetics.

[39] Lindgren G (2004). Limited number of patrilines in horse domestication. Nat. Genet..

[40] Parma P, Erra-Pujada M, Feligini M, Greppi G, Enne G (2004). Water buffalo (*Bubalus bubalis*): Complete nucleotide mitochondrial genome sequence. DNA Seq..

[41] Olivieri A (2015). Mitogenomes from Egyptian cattle breeds: new clues on the origin of haplogroup Q and the early spread of *Bos taurus* from the Near East. PLoS One.

[42] Eltsov, N. P. & Volodko, N. V. In http://eltsov.org (2011).

[43] Achilli A (2008). Mitochondrial genomes of extinct aurochs survive in domestic cattle. Curr. Biol..

[44] Lancioni H (2013). Phylogenetic relationships of three Italian merino-derived sheep breeds evaluated through a complete mitogenome analysis. PLoS One.

[45] Tamura K, Stecher G, Peterson D, Filipski A, Kumar S (2013). MEGA6: Molecular Evolutionary Genetics Analysis Version 6.0. Mol. Biol. Evol..

[46] Yang Z (2007). PAML 4: Phylogenetic Analysis by Maximum Likelihood. Mol. Biol. Evol..

[47] Drummond AJ, Rambaut A (2007). BEAST: Bayesian Evolutionary Analysis by Sampling Trees. BMC Evol. Biol..

[48] Drummond AJ, Rambaut A, Shapiro B, Pybus OG (2005). Bayesian coalescent inference of past population dynamics from molecular sequences. Mol. Biol. Evol..

[49] Bollongino R (2012). Modern Taurine Cattle descended from small number of Near-Eastern founders. Mol. Biol. Evol..

