# Supplementary material for: Whole Mitogenomes Reveal the History of Swamp Buffalo: Initially Shaped by Glacial Periods and Eventually Modelled by Domestication
Source: Sci Rep. 2017 Jul 5;7:4708. doi: 10.1038/s41598-017-04830-2 (PMC5498497; doi:10.1038/s41598-017-04830-2)
Supplement: Supplementary file 1 — Supplementary Figures S1-S6 and Tables S1-S2 [file 41598_2017_4830_MOESM1_ESM.pdf]

Supplementary Figures S1-S6 and Tables S1-S2

Whole Mitogenomes Reveal the History of Swamp Buffalo: Initially Shaped by Glacial Periods and Eventually Modelled by Domestication

S. Wang[1+], N. Chen[1+], M.R. Capodiferro[2+], T. Zhang[3], H. Lancioni[4], H. Zhang[5], Y. Miao[6], V. Chanthakhoun[7], M. Wanapat[8], M. Yindee[9], Y. Zhang[10], H. Lu[3], L. Caporali[11], R. Dang[1], Y. Huang[1], X. Lan[1], M. Plath[1], H. Chen[1], J. A. Lenstra[12], A. Achilli[2+]*, C. Lei[1+]*

1. College of Animal Science and Technology, Northwest A&F University, Yangling, Shaanxi 712100, China

2. Dipartimento di Biologia e Biotecnologie "L. Spallanzani", Università di Pavia, Pavia, 27100, Italy

3. School of Bioscience and Engineering, Shaanxi University of Technology, Hanzhong, Shaanxi 723000, China

4. Dipartimento di Chimica, Biologia e Biotecnologie, Università di Perugia, Perugia, 06123, Italy

5. Key Laboratory of Plateau Lake Ecology and Global Change, College of Tourism and Geography, Yunnan Normal University, Kunming, Yunnan 650500, China

6. Faculty of Animal Science and Technology,Yunnan Agricultural University, Kunming, Yunnan 650201, China

7. Department of Animal Science, Faculty of Agriculture and Forest Resource, Souphanouvong University, Luang Prabang, Laos

8. Tropical Feed Resources Research and Development Center, Department of Animal Science, Faculty of Agriculture, Khon Kaen University, Khon Kaen 40002, Thailand

9. Department of Clinical Science and Public Health, Faculty of Veterinary Science, Mahidol University, Kanchanaburi campus, Kanchanaburi 71150, Thailand.

10. National Engineering Laboratory for Animal Breeding, Key Laboratory of Aniaml Genetics and Breeding and Reproduction of MOA, College of Animal Science and Technology, China Agricultural University, Beijing 100193, China

11. IRCCS Institute of Neurological Sciences of Bologna, Bologna, 40139, Italy

12. Faculty of Veterinary Medicine, Utrecht University, Yalelaan 104, 3584 CM Utrecht, The Netherlands

+These authors contributed equally to this work

***Corresponding author:** alessandro.achilli@unipv.it; Dipartimento di Biologia e Biotecnologie "L. Spallanzani", Via Ferrata 9, Università di Pavia, Pavia, 27100, Italy; tel: +39 0382 985560. leichuzhao1118@126.com; College of Animal Science and Technology, Northwest A&F University, Yangling, Shaanxi 712100, China.

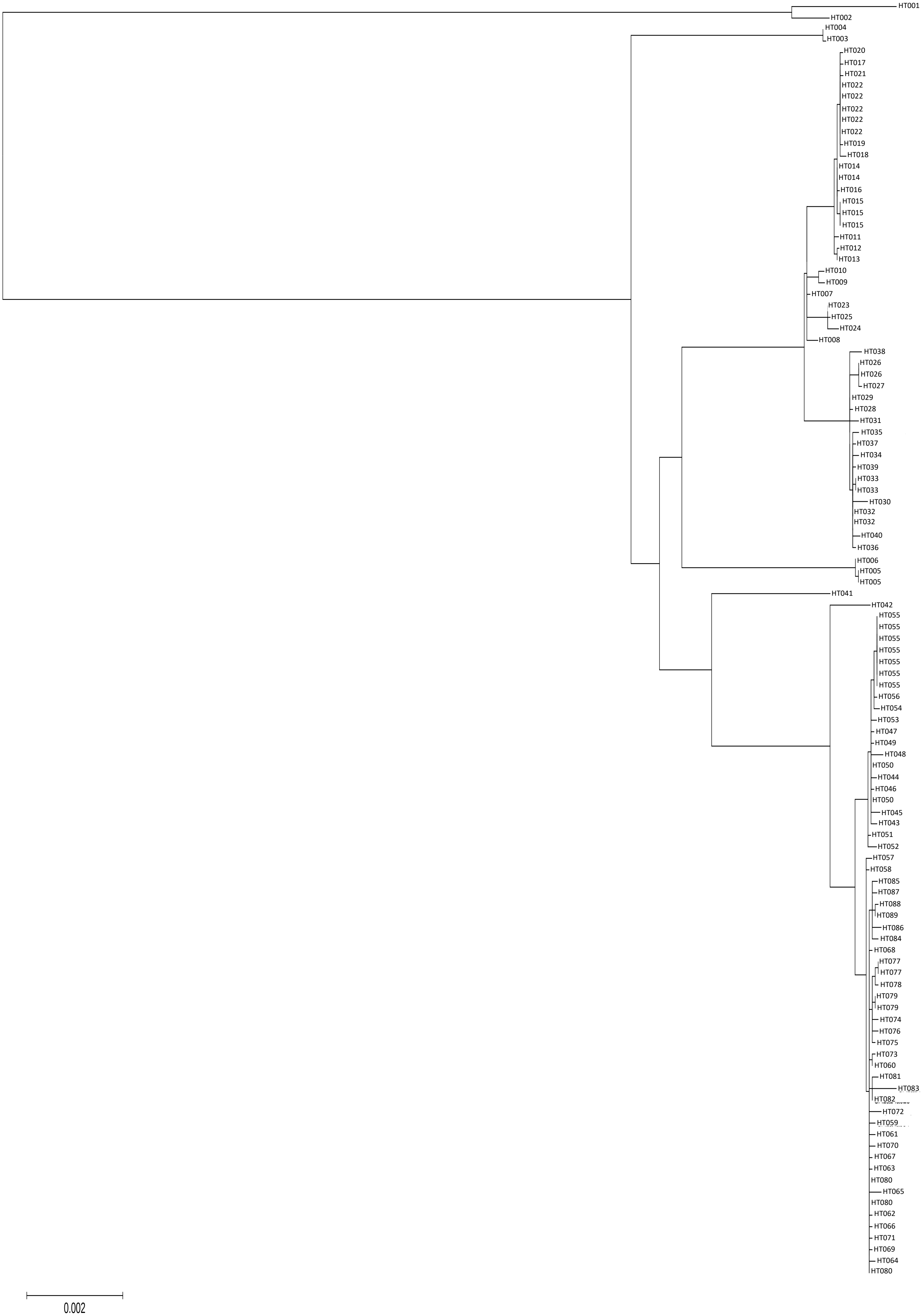

Fig. S1. The reconstructed phylogenies assessed with Mega (ML algorithm).

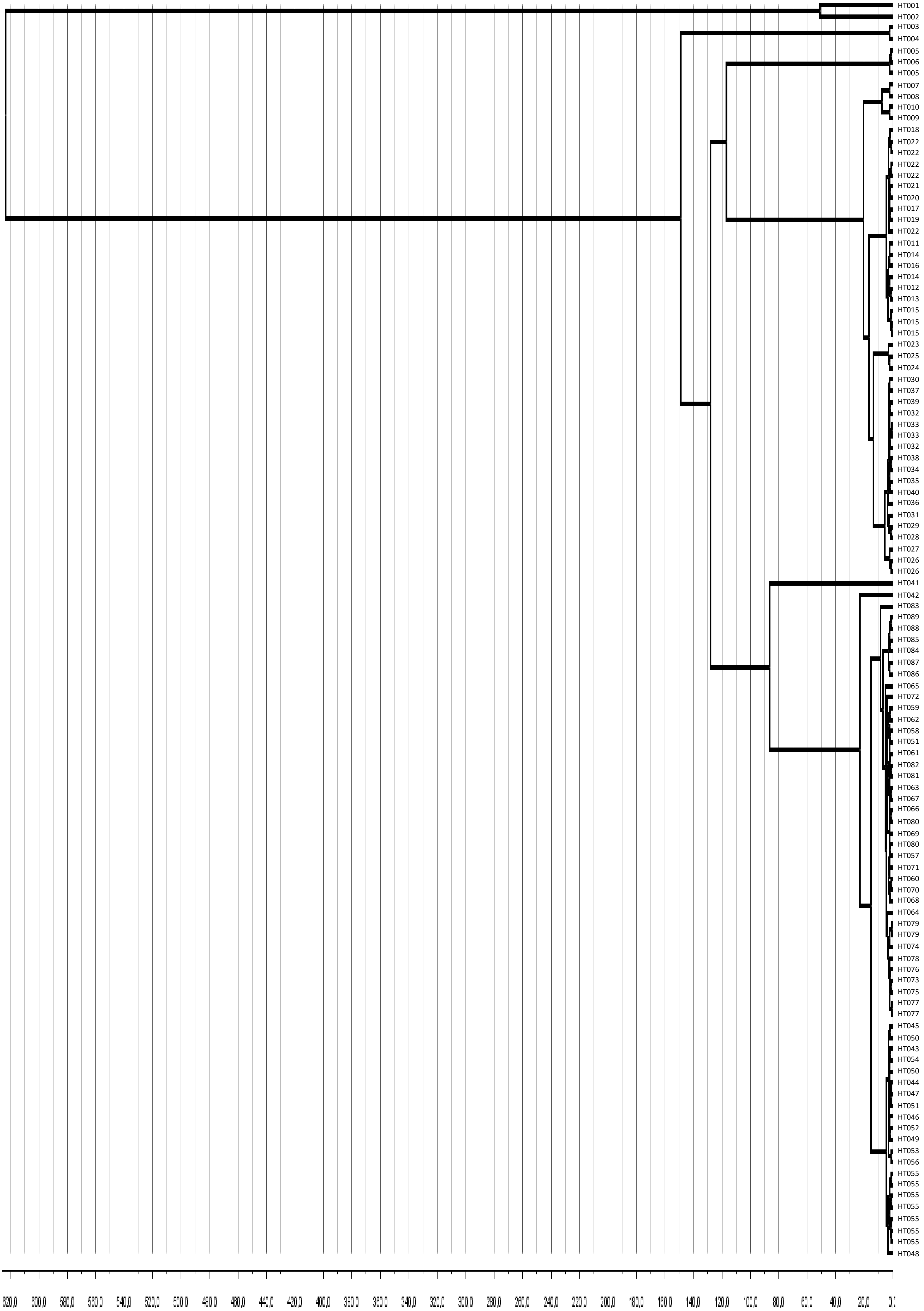

Fig. S2. The reconstructed phylogenies assessed with Beast.

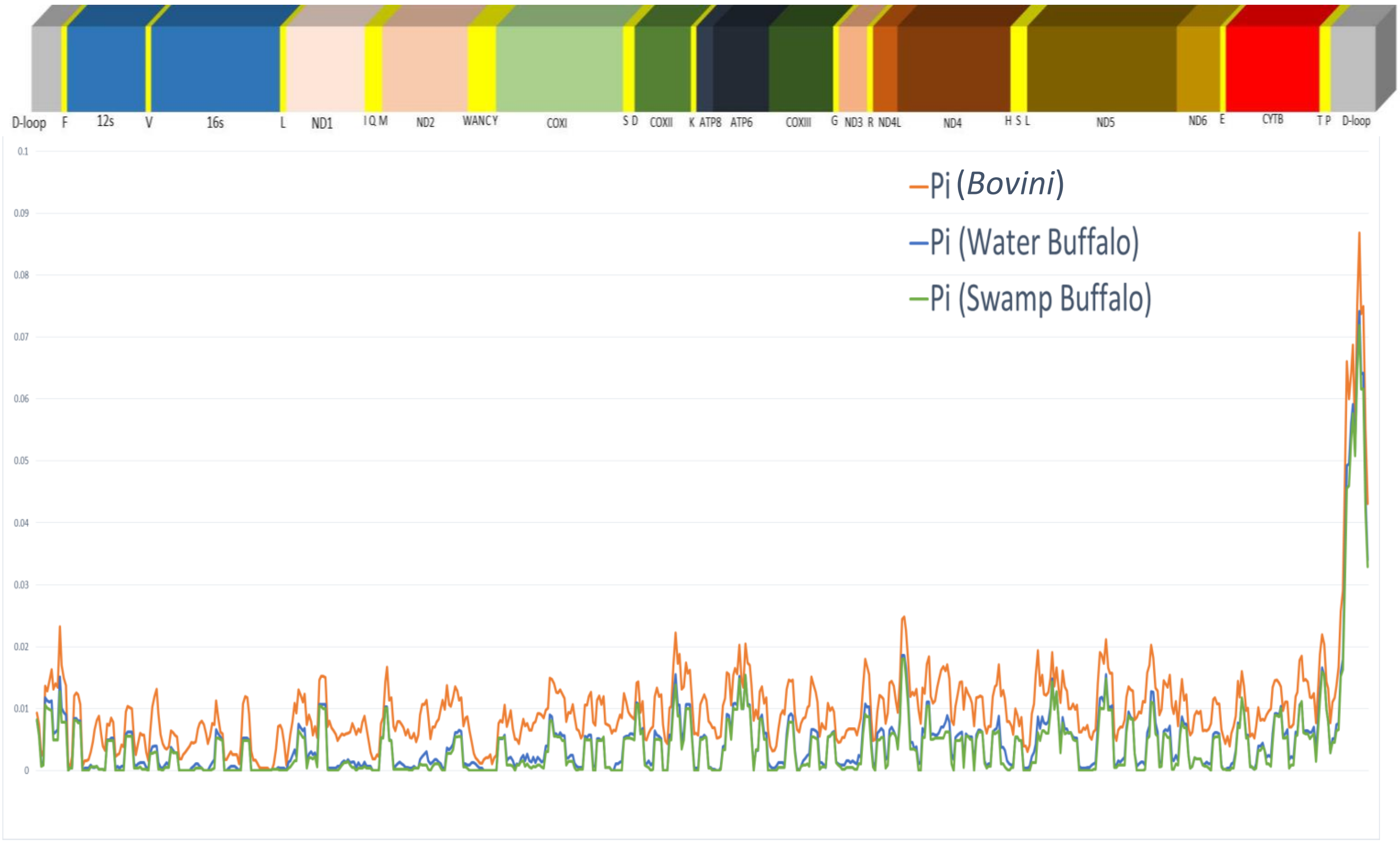

Fig. S3. Linearized gene map and nucleotide diversity along the entire mtDNA by considering windows of 200 bps centred in the midpoint.

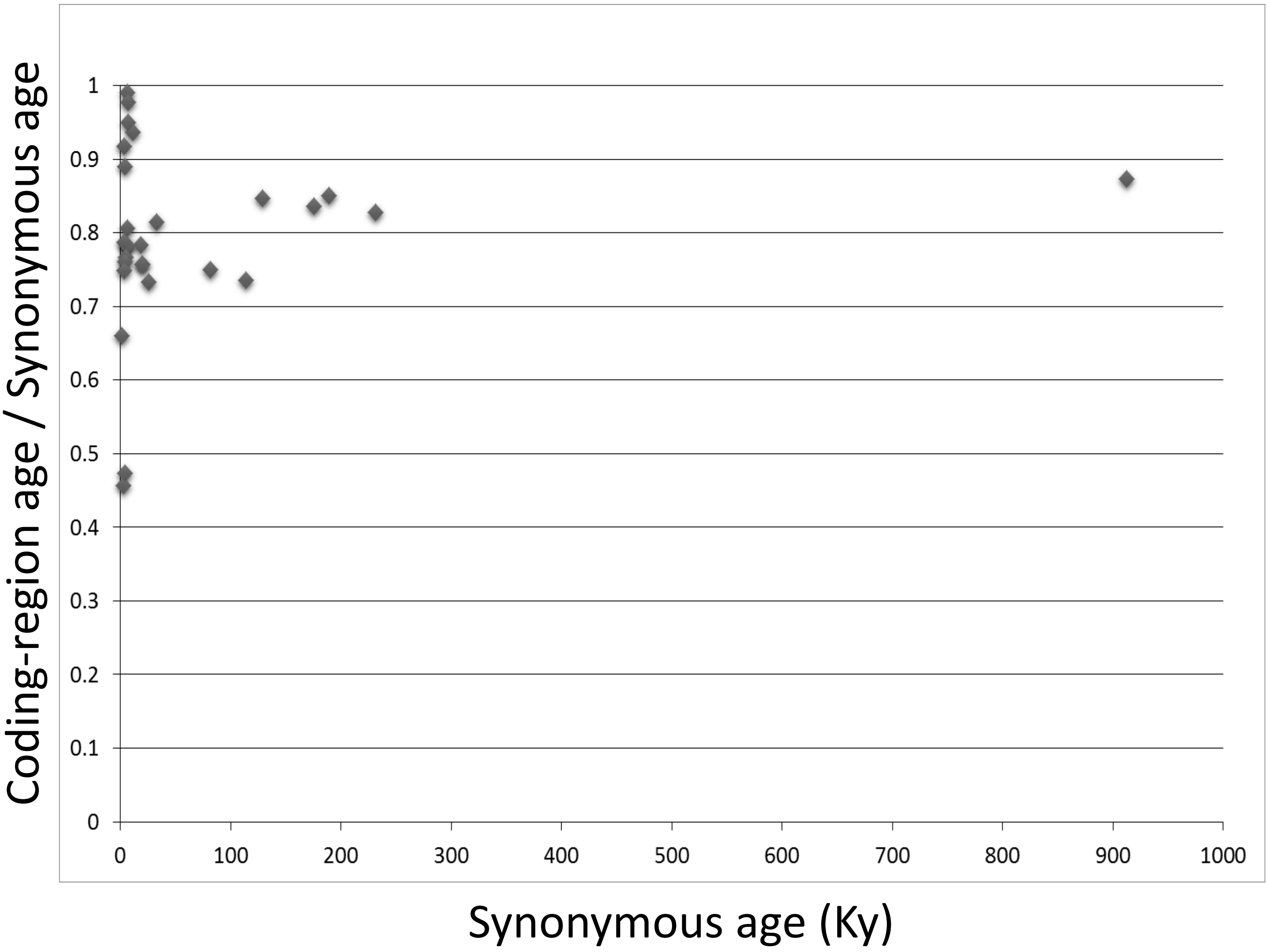

Fig. S4. Comparison of coding-region ages relative to the synonymous estimates (CodeML). See Table 1 for further details.

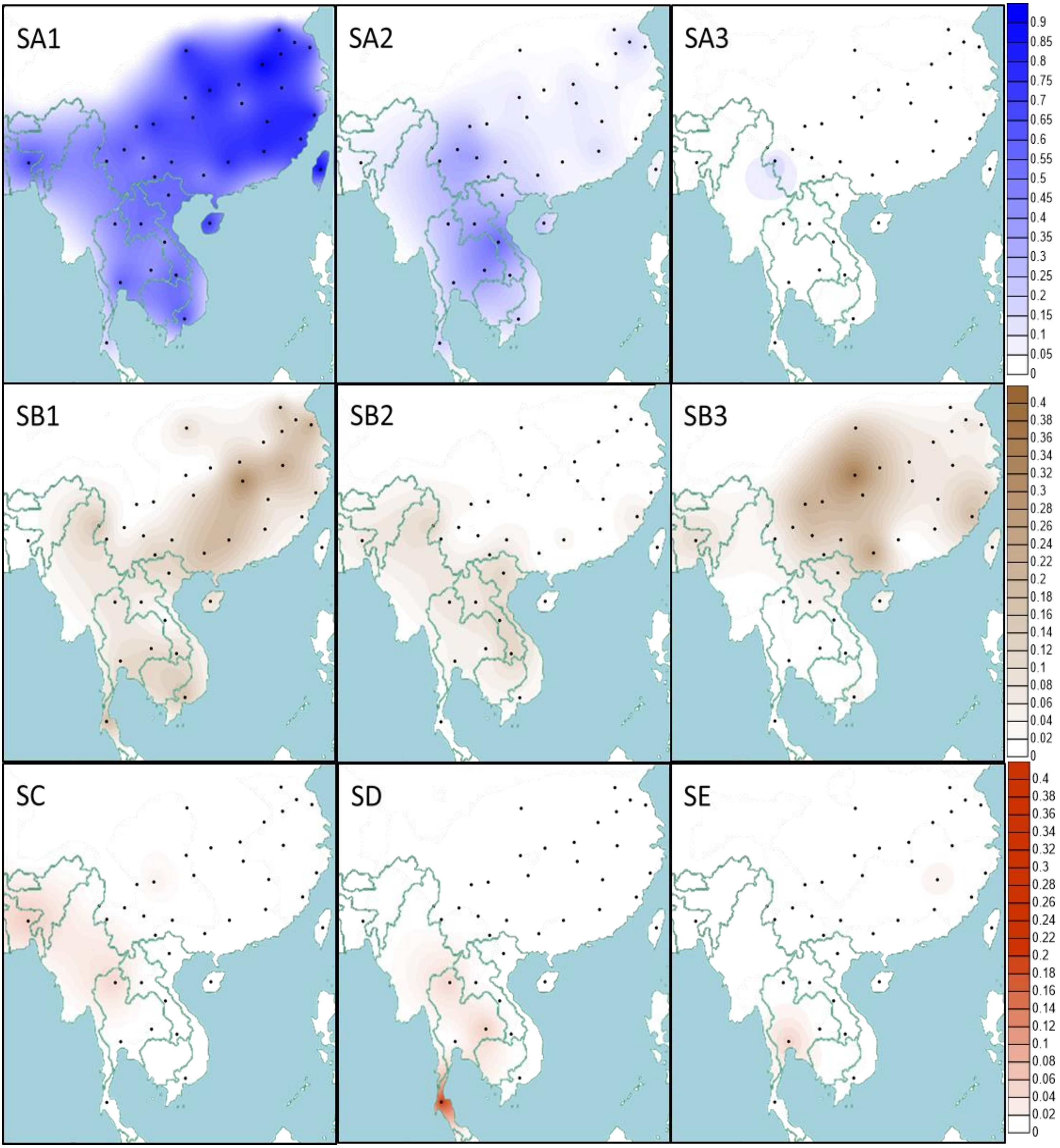

Fig. S5. Spatial frequency distributions of swamp buffalo mtDNA haplogroups in different geographic areas based on previously published control-region data (Supplementary Table S1) and integrated with our complete mitogenome dataset (Supplementary Dataset S1). Maps were generated with Surfer 9 (Golden Software, http://www.goldensoftware.com/products/surfer).

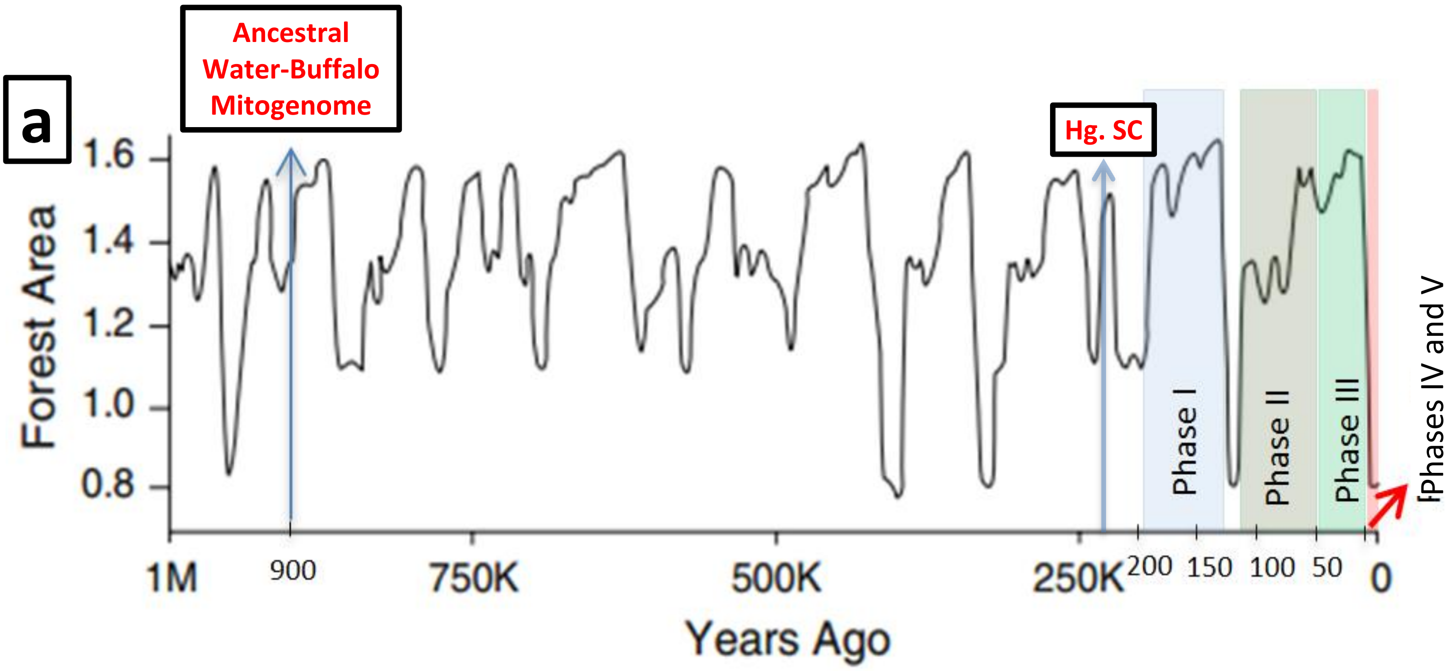

Fig. S6. Proposed demographic phases and forest area fluctuations in Southeast Asia during the last one million years (My), as reported in Woodruff 2010 [28].

Table S1. Haplogroup frequencies of swamp buffalo based on mtDNA control-region.

Country/ reigon	Population	Code	N	Lineages									N (SNPs)	N (Ht)	H^a	π^b
				SA1	SA2	SA3	SB1	SB2	SB3	SC	SD	SE				
China	Dangshan	C_DS	36	0.722	0.056		0.167	0.028	0.028				57	24	0.941	0.01487
	Fengyang	C_FY	53	0.792	0.038		0.132		0.038				52	22	0.835	0.01166
	Shanqu	C_SQ	30	0.633	0.167		0.133		0.067				44	12	0.800	0.01342
	Haizi	C_HZ	50	0.640	0.080		0.220		0.060				60	26	0.904	0.01738
	Xinyang	C_XY	29	0.897			0.034		0.069				47	12	0.663	0.00810
	Dongliu	C_DL	49	0.673	0.041		0.224		0.061				51	23	0.893	0.01674
	Binhu	C_BH	37	0.568	0.054		0.324		0.054				48	16	0.899	0.01892
	Jianghan	C_JH	61	0.656	0.098		0.164		0.082				57	30	0.938	0.01604
	Xiajiang	C_XJ	39	0.821	0.077		0.077		0.026				51	18	0.808	0.00856
	Xinfeng	C_XF	24	0.750	0.125		0.083		0.042				45	10	0.746	0.01005
	Fuan	C_FA	42	0.690			0.048	0.071	0.190				52	18	0.876	0.01837
	Wenzhou	C_WZ	30	0.767			0.100		0.133				50	14	0.834	0.01543
	Taiwan	C_TW	29	0.897	0.069				0.034				41	8	0.828	0.00430
	Enshi	C_ES	45	0.800					0.200				44	17	0.844	0.01356
	Fuling	C_FL	27	0.593	0.074				0.333				47	16	0.906	0.01950
	Guizhou	C_GZ	54	0.648	0.074		0.074		0.204				70	25	0.906	0.01796
	Shannan	C_SN	51	0.745	0.020		0.098		0.137				53	20	0.805	0.01532
	Fuzhong	C_FZ	30	0.767	0.033		0.167	0.033					47	11	0.851	0.01333
	Guangxi	C_GX	13	0.462	0.152		0.152		0.231				47	9	0.923	0.02151
	Xinlin	C_XL	42	0.571	0.167		0.095	0.048	0.119				51	19	0.875	0.01692
	Yanjin	C_YJ	29	0.724	0.069				0.207				44	10	0.805	0.01472
	Dechang	C_DC	36	0.528	0.222			0.028	0.222				49	16	0.917	0.01675
	Dali	C_DA	25	0.560	0.320				0.120				40	10	0.847	0.01073
	Ershan	C_ER	25	0.480	0.400				0.120				42	11	0.887	0.01090
	Diandongnan	C_DD	54	0.537	0.167		0.130	0.074	0.093				60	28	0.895	0.01830
	Xinglong	C_XG	58	0.707	0.190		0.069		0.034				55	22	0.834	0.00951
	Dehong	C_DH	70	0.486	0.300	0.057	0.071	0.043	0.029	0.014			83	34	0.899	0.01377
	Binlangjiang	C_BL	24	0.500	0.167		0.167	0.125	0.042				53	15	0.942	0.01983
Vietnam	Ha Giang	VN_N	75	0.533	0.173		0.120	0.107	0.067				63	37	0.929	0.01785
	Tay Ninh	VN_S	25	0.680	0.200		0.040	0.040	0.040				46	13	0.810	0.01044
Laos	Xayaboury	LA_N	39	0.487	0.436			0.077					47	17	0.910	0.00871
	Khammouane	LA_C	17	0.353	0.529			0.118					42	10	0.904	0.01224
	Champasak	LA_S	40	0.550	0.225		0.100	0.125					48	13	0.847	0.01558
Thailand	North	TH_N	11	0.455	0.273		0.091	0.091		0.091			55	8	0.891	0.01928
	Northeast	TH_NE	18	0.500	0.222		0.167	0.056			0.056		57	11	0.928	0.01888
	Central	TH_C	20	0.550	0.250		0.100	0.050				0.050	58	14	0.953	0.01567
	South	TH_S	5	0.200	0.200		0.200				0.400		55	5	1.000	0.03234
Bangladesh	Bangladesh	BD	21	0.714	0.048			0.095		0.143			53	7	0.714	0.01467
	**TOTAL**		**1363**	**0.640**	**0.138**	**0.003**	**0.098**	**0.031**	**0.084**	**0.004**	**0.002**	**0.001**				

Sequences that were used (1363 total):
DQ364160-DQ364189 and DQ658051-DQ658139 [1]
GQ260217–GQ260323, GQ260340-GQ260455 [2]
913 D-loop sequences of swamp buffalo [3]
AY702618, EF597573-EF597647, EF597653-EF597662 and EF053531-EF053552
[a] Haplotype diversity; [b] nucleotide diversity.

[1] Lei C Z, Zhang W, Chen H, et al. Independent maternal origin of Chinese swamp buffalo (*Bubalus bubalis*)[J]. Animal Genetics, 2007, 38(2): 97-102.
[2] Yue X P, Li R, Xie W M, et al. Phylogeography and domestication of Chinese swamp buffalo[J]. PloS one, 2013, 8(2): e56552.
[3] Zhang Y, Lu Y, Yindee M, et al. Strong and Stable Geographic Differentiation of Swamp Buffalo Maternal and Paternal Lineages Indicates Domestication in the China/Indochina Border Region[J]. Molecular ecology, 2015.

Table S2A. Oligonucleotides used to amplify and sequence (Sanger method) the water buffalo mitochondrial genome.

Fragment #	PCR product length (bp)	Oligonucleotides 5'-3'		Tm (℃)
1	744	B1 F	GATCACGAGCTTGGTCACCA	59.00
		B1 R	ATGCCCGCTCCTCTTAGTTT	
2	652	B3 F	CCGTCACCCTCCTCAAGTAA	59.00
		B3 R	GGTATCCGTTTCTAAAAGGCTG	
3	693	B2 F	TCACATGGATTGGAGGACAG	57.00
		B2 R	ATCCCTGTCTGAAGGGTTG	
4	665	B4 F	CGCACGAGGGTTTTACTGTC	57.00
		B4 R	AAGGAGAGGATTTGAATCTCTG	
5	913	B5 F	AGTCTCAGGCTTCAACGTAG	57.00
		B5 R	GGGATACCTTGTGTTACTTC	
6	1079	B8 F	ATCGGAGGAGCTACACTTGC	53.00
		B8 R	AATGCGATGATAACAAGTA	
7	776	B10 F	TCACTTATCCAGATGAAAGC	53.00
		B10 R	AGCAGGGAGGTCAATGAATG	
8	965	B9 F	TTTACACGGGAAAATGCACT	51.50
		B9 R	GGATTTTCCTGTTGCGGCTA	
9	676	B11 F	GCTGGTTGTCCAGAAAATGAA	51.50
		B11 R	CGGGAAGGTCAATTTCACTG	
10	1028	B12 F	AAGGTTCGTTTGTTCAACGATT	51.50
		B12 R	TATGGGTTGTGGGATGTTCC	
11	712	B13-1 F	GATATGGCATGCTACGAATC	56.50
		B13-1 R	GGGCTTCTATTGTTAGATTCAC	
12	941	B13-2 F	GCTGCCTGATATTGACACTTTG	56.50
		B13-2 R	GCTGTGGGCTGTAGAGTTAAT	
13	966	B13new F	TCATCTTACTGGTCTTCGCA	56.50
		B13new R	ATGAGGGATTTCAGGTCTGT	
14	769	B14-1 F	TCCAGATGAAAGCATCCATC	56.50
		B14-1 R	AGCAGGGAGGTCAATGAATG	
15	893	B14-2 F	CAAACACAGCAGCCCTACAA	56.50
		B14-2 R	ACTTCCGATTAGCAAGCGTT	
16	830	B14new F	ACCTCATTCACAGCCATCTA	56.50
		B14new R	TAAAGTTGTCTTAGGGGCGT	
17	790	B17-1 F	TCTACATACGACTCGCATACTCC	56.50
		B17-1 R	GTCAGTTGCCAAATCCCCCA	
18	779	B17-2 F	GGCCAATGGACCGTAATAAA	56.50
		B17-2 R	GGTGTTAGTGGTAGTAGTATGG	
19	792	B18-1 F	GCCACAGTTAGACACATCAAC	56.50
		B18-1 R	GGCTTGGATTATAGCCACTGC	
20	745	B18-2 F	GCCCTAGGCTTTATCTTCCT	56.50
		B18-2 R	TGGTCATGAAAGTGAAGCAG	
21	984	B18new F	TAAGTTACAAGTGAGAACCCTG	56.50
		B18new R	GGCGTAATGAAAGAGGCAAATA	
22	971	B22 F	GAAAATGCGTAGATGAGTTC	56.50
		B22 R	TATGCTTACCTTGTTACGAC	
23	599	Cytb-1 F	AACAGGATCCAACAACCCAAC	56.50
		Cytb-1 R	GAGGTTGGTTGTTCTCCTTTTCTGG	
24	746	Cytb-2 F	ACCACGACCAATGATATGAAAAACC	56.50
		Cytb-2 R	CCTAGGATGTCTTTAATGGTGT	
25	755	B15-1 F	TAAATGCCCTAATCAACTGG	54.00
		B15-1 R	TCATACGAATAAGGGGGTTT	
26	800	B15-2 F	GGACCGTATACCCCCCTTTA	54.00
		B15-2 R	TCCTCCTATAATGGCGAACA	
D-loop	1160	CB1	TAGTGCTAATACCAACGGCC	55.50
		CB2	AGGCATTTTCAGTGCCTTGC	

Table S2B. Amplicons and oligonucleotides used for sequencing the whole mitochondrial genome with the Illumina MiSeq®.

Fragment #	PCR product length (bp)	Oligonucleotides 5'-3' [a]		Tm
1	8810	3921 For	CCTTTCTATGAATCCGAGCATCC	59.00
		12730 Rev	GAAGATTTGTTGGAGGTCTCAGG	58.99
2	8357	12708 For	CCTGAGACCTCCAACAAATCTTC	58.99
		4705 Rev	AGTACGGATATGGGTGCTAGTTT	59.03

[a] Primer names correspond to the (5') nucleotide position in the buffalo reference sequence (NC006295); For, forward; Rev, reverse.
